# Efficacy of free glutathione and niosomal glutathione in the treatment of acetaminophen-induced hepatotoxicity in cats

**Published:** 2013-06-08

**Authors:** L.A. Denzoin Vulcano, O. Confalonieri, R. Franci, M.O. Tapia, A.L. Soraci

**Affiliations:** 1 *Department of Physiopathology, Faculty of Veterinary Science, UNCPBA, Campus Universitario, Paraje Arroyo Seco. Tandil, 7000, Argentina*; 2 *Department of Clinic, Faculty of Veterinary Science, UNCPBA, Campus Universitario, Paraje Arroyo Seco. Tandil, 7000, Argentina*

**Keywords:** Acetaminophen toxicity, Cats, Glutathione, Niosomes

## Abstract

Acetaminophen (APAP) administration results in hepatotoxicity and hematotoxicity in cats. The response to three different treatments against APAP poisoning was evaluated. Free glutathione (GSH) (200mg/kg), niosomal GSH (14 mg/kg) and free amino acids (180 mg/kg of N-acetylcysteine and 280 mg/kg of methionine) were administered to cats that were intoxicated with APAP (a single dose of 150 mg/kg, p.o.). Serum concentration of alanine aminotransferase (ALT) along with serum, liver and erythrocyte concentration of GSH and methemoglobin percentage were measured before and 4, 24 and 72 hours after APAP administration. Free GSH (200 mg/kg) and niosomal GSH (14 mg/kg) were effective in reducing hepatotoxicity and hematotoxicity in cats intoxicated with a dose of 150 mg/kg APAP. We conclude that both types of treatments can protect the liver and haemoglobin against oxidative stress in APAP intoxicated cats. Furthermore, our results showed that treatment with niosomal GSH represents an effective therapeutic approach for APAP poisoning.

## Introduction

Acetaminophen (APAP) is one of the most widely used analgesic and antipyretic in human medical practice (Mirochnitchenko *et al.*, 1999). However, APAP is highly toxic to cats and dogs when administered at human doses. The common factor in APAP poisoning is the erroneous administration by the owner without prior consultation with the veterinarian. For this reason, APAP intoxication represents a common cause of feline and canine toxicoses reported to veterinary poison treatment centers (Jones *et al.*, 1992; Allen, 2003).

In particular, there is no safe dose of APAP for cats. Studies have shown that a dose of 50 to 100 mg/kg bodyweight of APAP is toxic to cats (Sellon, 2001). There is also evidence which indicates that a dose as low as 10 mg/kg bodyweight can produce signs of toxicity and lead to death (Aronson and Drobatz, 1996). The pathways for APAP metabolism are via glucuronidation and sulfation, followed by renal excretion of the conjugates in most of the mammalian species (Davis *el al*., 1974; Savides *et al.*, 1984).

A small percentage of APAP is oxidized by cytochrome P450 enzymes (metabolic activation of APAP) to the highly reactive metabolite, N-acetyl-p-benzoquinoneimine (NAPQI) (Davis *et al.*, 1974). NAPQI binds to glutathione (GSH) and is excreted in the form of mercapturic acid conjugate (Davis *et al.*, 1974).

The domestic cat (*Felis catus*) is extremely sensitive to the toxic effects of APAP for several reasons. One of the main reasons is slow and poor glucuronidation ability in this species. Poor glucuronidation is due to UDP glucuronosyltransferase 1A6 (UGT1A6), the key isoenzyme responsible for APAP detoxification, being not expressed in the liver of cats.

In the Felidae family, lack of UGT1A6 isoenzyme is the result of multiple inactivating mutations of the gene that encodes UGT1A6, rendering the species defective for APAP metabolism (Court and Greenblatt, 2000; Shrestha *et al.*, 2011). Another reason for APAP toxicity is the limited capacity of the sulfation pathway in cats compared to other species (Savides *et al.*, 1984). For both the reasons mentioned above, APAP persists in the blood and as a result, APAP is metabolized by cytochrome P-450 enzymes to the highly reactive metabolite NAPQI which further depletes the GSH reserves (Allen, 2003).

Another detrimental effect of NAPQI is that it reacts with cellular proteins and affects mitochondrial respiration (early mitochondrial injury). NAPQI-induced oxidative stress also leads to a delayed mitochondrial injury (Jaeschke *et al.*, 2011) and cell death. Erythrocytes are the cells that are most susceptible to the toxic effects of NAPQI in the cats (McConkey *et al.*, 2009).

Two key components in erythrocytes are susceptible to NAPQI-induced oxidative injury: the iron in heme and the sulfhydryl groups of the globulin chains. NAPQI causes the oxidation of ferrous iron (Fe_2_+) to ferric iron (Fe_3_+), which converts hemoglobin to methemoglobin (Richardson, 2000). In this regard, it is important to note that cats have a relative deficiency of methemoglobin reductase in erythrocytes. Thus, methemoglobinemia is a primary and prominent feature of APAP toxicity in this species.

Another deleterious effect of NAPQI in cats is the oxidation of thiol groups of hemoglobin with the consequent production of large amounts of methemoglobin and Heinz bodies (Desnoyers, 2000). This particular reaction in cats has been attributed to the high numbers of sulfhydryl groups that are exposed on the surface of feline haemoglobin.

Traditional treatment of APAP toxicity involves the administration of alternate sulfhydryl donors in order to inhibit the formation of the reactive metabolite (Savides *et al.*, 1985). N-acetylcysteine (NAC) is the most widely used compound for the treatment of APAP toxicity (Polson and Lee, 2005). This compound protects against oxidative injury by providing cysteine groups for GSH biosynthesis and also by directly inactivating NAPQI via its sulfhydryl group (Corcoran and Wong, 1986).

In contrast to NAC, GSH has not been used clinically in APAP poisoning because it is rapidly hydrolyzed and has a short plasma half-life ranging from 2 to 15 minutes (Wendel and Jaeschke, 1982; Hong *et al.*, 2005; Saito *et al.*, 2010). However, recent studies indicate that intravenous administration of GSH was effective in treating mice exhibiting APAP toxicity, because it causes an increase in the levels of hepatic GSH due to *de novo* synthesis from amino acids released by the hydrolysis of GSH (Knight *et al.*, 2002; Saito *et al.*, 2010; Masubuchi *et al.*, 2011).

Niosomal GSH could be effective in the treatment of APAP intoxication. Encapsulation into vesicles protects the tripeptide from being hydrolysed and thereby permits a higher concentration to reach the liver. Niosomes (non–ionic surfactant vesicles) are nanovesicular drug delivery systems formed by self-assembly of molecules of nonionic surfactant and cholesterol. The surfactants used and also the prepared niosomes are biodegradable, biocompatible and non-immunogenic (Sankhyan and Pawar, 2012).

These vesicles are used in the transport of active compounds in the form of drugs, genes or vaccine fractions. Niosomes can modify the pharmacokinetics and bioavailability of the drugs (Baillie *et al.*, 1985; Uchegbu and Florence, 1995). After intravenous administration, the biological behavior (pharmacokinetics, arrival at the site of action, therapeutic efficacy and adverse reactions) of the niosomes is controlled by a complex set of physicochemical and biological factors that include the size of the vesicles, shape and composition of the membrane, biochemical, anatomical and immunological barriers (Moghimi *et al.*, 2012).

Thus, not only the characteristics, but also the performance of the prepared niosomes can be exquisitely controlled by altering the composition, concentration of various additives, size, lamellarity and surface charge of vesicles (Khan *et al.*, 2011). Studies show that the therapeutic efficacy of the drugs can be significantly improved by reducing the clearance rate, targeting to the specific site and by protecting the encapsulated drug (Sankhyan and Pawar, 2012).

The purpose of this study was to compare the efficacy of NAC, free GSH and niosomal GSH in the treatment of APAP toxicity in cats.

## Material and Methods

### Drugs and reagents

Reduced L-GSH, O-phthaldialdehyde (OPA), DL-dithiothreitol (DTT), tris hydroxymethyl aminomethane and sodium tetraborato, dihexadecyl phosphate (DCP) were obtained from Sigma Aldrich (St Louis, USA). Sodium acetate was obtained from Farmitalia, Carlo Erba (Milano, Italy). Sodium dihydrogen phosphate monohydrate, cholesterol and chlorhydric acid were obtained from JT Baker (Xalostos, Mexico).

Methanol and acetonitrile HPLC grade were obtained from JT Baker (Phillipsburg, USA). Metaphosphoric acid (MPA) was obtained from Riedel-de Haën (Seelze, German); ethylenediaminetetraacetic acid solution 0.342 mol/l (EDTA) was obtained from Wiener Lab, Argentina. APAP (®Paracetamol Raffo, 1 g); ALT UV AA diagnostic kit (Wiener Lab, Argentina); hemoglobin standart (Wiener Lab, Argentina); potassium ferricyanide (Biopack, Argentina); potassium cyanide (Biopack, Argentina). Sorbitol monostearate (Span 60) was obtained from Fluka, Spain; polycarbonate membranes (Isopore) of 0.8, 0.4 and 0.2 µm were obtained from Millipore. Saline Sodium Chloride 0.9% was obtained from Roux-Ocefa U (Argentina).

### Preparation of GSH niosomes

Preparation of niosomes was carried out at ~65°C. It was essential to prepare the vesicles at a temperature above the gel-liquid transition temperature of span 60. Niosomes were prepared by hydration of melted surfactant/cholesterol/DCP (67.5: 27.5: 5) at 65°C in a 50 ml beaker (Niemiec *et al.*, 1995; Uchegbu and Vyas, 1998; Confalonieri *et al.*, 2010). The non-ionic surfactant was melted using a magnetic stirrer with a heated plate (AREC, Velp Scientifica), once melted, cholesterol and DCP were added and the mixture was hydrated with a GSH solution in saline sodium chloride (5.4 M) by shaking with the magnetic stirrer for 60 min. Then the suspensions were processed for 3 min at 16,000 rpm (Ultraturrax T-25) and finally, sonicated for 3 minutes at 40 Khz power (Mod Testlab Ultrasound TBO10). Once this process was completed the suspensions were maintained at 65°C and manually extruded, five times each, successively through polycarbonate membranes (0.8, 0.4 and 0.2 μm).

### Determination of GSH entrapment efficiency

The removal of the unbound GSH from the vesicles was accomplished by ultracentrifugation at 150,000 g at 4°C for 90 minutes (Ultracentrifuge Sorvall Ultra Pro 80). The isolated pellets were washed twice, each with saline sodium chloride, and recentrifuged again for 1 hour. The supernatant was removed and the pellets were resuspended in the saline sodium chloride to obtain a total lipid concentration of 20 mM.

The amount of entrapped GSH was determined after disruption of vesicles by lipid extraction method (Bligh and Dyer, 1959). The GSH incorporated into the niosomes was analysed by HPLC as described previously (Denzoin *et al.*, 2008). Entrapment efficiency (E%) was expressed as a percentage of the total amount of GSH found in the disrupted vesicles. The entrapment efficiency of GSH is calculated as follows (Ruckmani *et al.*, 2000):


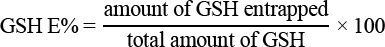


### Characterization of GSH niosomes

Vesicles were characterised by optical and light polarized microscopy (Olympus BX40) for vesicle formation, agglutination and morphology. Samples of the niosomal formulations were examined under the optical microscope and were photographed at a magnification of 1000×. The size distribution and the polydispersivity index of the vesicles was determined using dynamic light scattering at 25°C with a scattering angle of 90° (Zetasizer Nano ZS, Malvern).

### Animals

Sixteen healthy adult, domestic shorthaired cats were used for this study. The mean age of the cats was 3.2 years old (range 2-5 years), and the average body weight was 3.84 ± 0.69 kg.

The cats were fed with cat food for adult maintenance (Association of American Feed Control Officials) on an *ad libitum* basis from 8:00 am to 6:00 pm daily. Water was also provided *ad libitum*. The animals were kept fasting for 12 hours prior to starting the experimental protocol. This study was performed according to the standards established by the Animal Welfare Commission of Faculty of Veterinary Science.

### Experimental protocol

The animals were divided in four groups containing four animals each (Groups I, II, III and IV) and anesthetized using a combination of ketamine (15 mg/kg) and xylazine (0.5 mg/kg). An intravenous catheter (Abbocath G18) was placed in the jugular vein to facilitate administration of drugs used for the treatment and an intravenous catheter (Abbocath G22) was placed in antebrachial cephalic vein for sampling the blood.

After recovery from the anaesthesia, the animals were transferred back to their cages. Group I (control) received a single dose of 150 mg/kg of APAP administrated orally. This dose was established by us in a previous work (Castro, 2001). Group II received a single oral dose of 150 mg/kg of APAP and a dose of GSH 200 mg/kg (mouse dose according to Wendel and Jaeschke (1982)) solubilized in 100 ml of physiological saline solution by intravenous infusion.

Group III received a single oral dose of 150 mg/kg APAP and a dose of niosomal GSH 14 mg/kg solubilized in 100 ml of physiological saline solution by intravenous infusion. This was the maximum dose of GSH that could be encapsulated into the niosomes. Group IV received a single oral dose of 150 mg/kg of APAP and an oral dose of 180 mg/kg of N-acetylcysteine and 280 mg/kg of methionine dissolved in 10 ml of physiological saline solution of sodium chloride 0.9% using a probe K30 tube.

### Blood and liver sampling

Blood and liver samples were collected from all the animals at 0, 4, 24 and 72 hours after dosing. Blood samples (5 ml) were collected and immediately placed into refrigerated tubes containing EDTA as an anticoagulant.

Three liver biopsies of each animal were collected using Menghini’s technique (Menghini, 1958) using local anaesthesia. The liver biopsies were placed in chilled physiological saline solution. The percentage of methemoglobin was determined by colorimetry (Sato, 2005).

Levels of Alanine animotransferase (ALT) was measured at 37°C in spectrophotometer (Pharmacia Ultrospec III) using the optimized method UV (Wiener Lab). GSH concentrations in plasma, erythrocytes and liver were determined according to the method previously described (Denzoin *et al.*, 2008). Liver protein concentration was determined using the Lowry *et al*. (1951) method.

### Statistical analysis

A completely randomized design was established with measurements repeated over time, using a linear mixed model with an autoregressive correlation structure of order 1 (InfoStat 2011p version).

## Results

### GSH niosomes

**Fig. 1:**
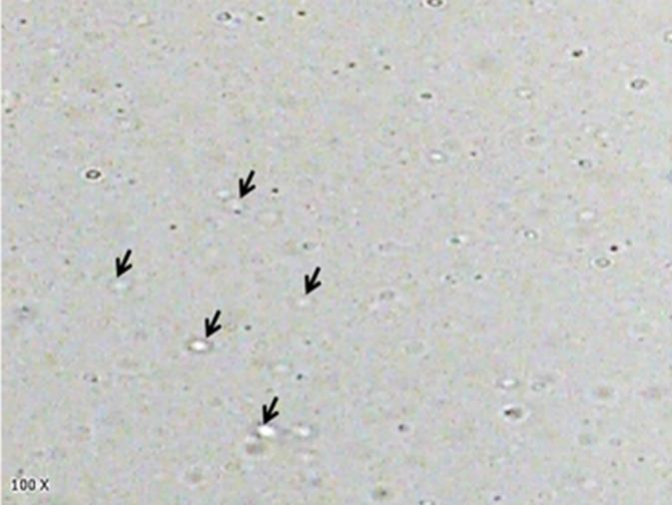
Photomicrograph of GSH loaded niosomes composed of Spam 60/cholesterol/DCP (67.5: 27.5: 5). The arrows show the vesicles.

The GSH entrapment efficiency of niosomes was 49.9 %. Photomicrographs of the GSH niosomes are shown in Fig. 1.

The GSH niosomes were spherical in shape. No aggregates were observed. The mean particle diameters were 422 nm.

### Clinical signs

Group I and Group IV exhibited clinical manifestations of APAP acute intoxication. Clinical characteristics included cyanosis and tachypnea between 2 and 6 hours post treatment followed by anorexia and depression between 24 and 72 hours. Groups II and III showed only anorexia. No deaths were recorded during the entire experimental protocol.

**Fig. 2:**
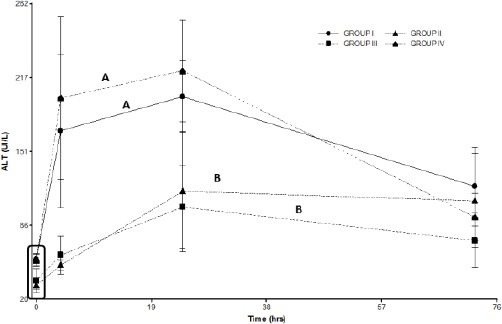
ALT levels in the treated groups. Group I: 150 mg/kg of APAP. Group II 150 mg/kg of APAPand GSH 200 mg/kg. Group III 150 mg/kg of APAPand niosomal GSH (14 mg/kg). Group IV 150 mg/kg of APAP, 180 mg/kg of N-acetylcysteine and 280 mg/kg of methionine. The square indicates the basal values. Different letters indicate significant differences between the groups (*p*<0.05).

Group IV showed no significant differences in ALT activity (*p*>0.05) compared to Group I. On the other hand, Group II and Group III showed a small but significant (*p*<0.05) increase in ALT levels when compared to Groups I and IV (Fig. 2).

**Fig. 3:**
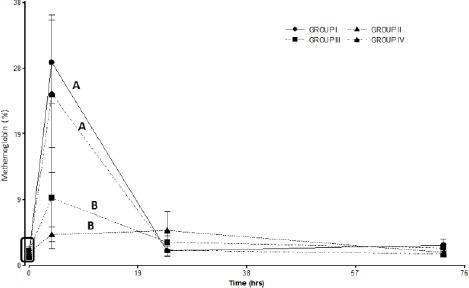
Methemoglobin percent in the treated groups. Group I: 150 mg/kg of APAP. Group II 150 mg/kg of APAPand GSH 200 mg/kg. Group III 150 mg/kg of APAPand niosomal GSH (14 mg/kg). Group IV 150 mg/kg of APAP, 180 mg/kg of N-acetylcysteine and 280 mg/kg of methionine. The square indicates basal values. Different letters indicate significant differences between the groups (*p*<0.05).

No significant differences in methemoglobin levels were observed between Groups I and IV. Groups II and III showed significant differences with respect to Groups I and IV (Fig. 3). Although no significant differences were observed between groups treated with free GSH and niosomal GSH, the group treated with free GSH (group II) showed lower methemoglobin formation than the group treated with niosomal GSH (group III).

**Fig. 4:**
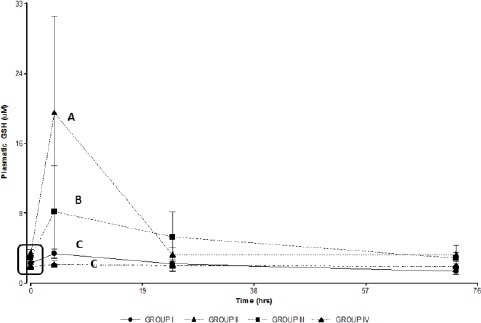
Plasmatic GSH concentrations in the treated groups. Group I: 150 mg/kg of APAP. Group II 150 mg/kg of APAPand GSH 200 mg/kg. Group III 150 mg/kg of APAPand niosomal GSH (14 mg/kg). Group IV 150 mg/kg of APAP, 180 mg/kg of N-acetylcysteine and 280 mg/kg of methionine. The square indicates basal values. Different letters indicate significant differences between groups (*p*<0.05).

There was a significant increase in plasma concentration of GSH at 4 hours post treatment in groups II and III when compared to the control group (group I). Likewise, significant differences in plasma GSH concentrations were found between groups II and III (Fig. 4).

**Fig. 5:**
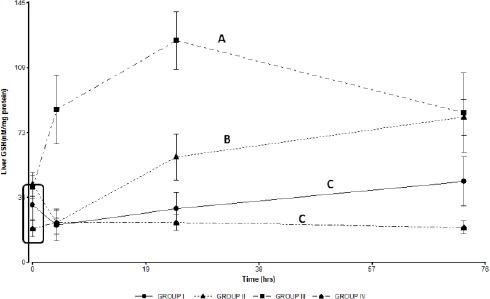
Liver GSH concentrations in the treated groups. Group I: 150 mg/kg of APAP. Group II 150 mg/kg of APAPand GSH 200 mg/kg. Group III 150 mg/kg of APAPand niosomal GSH (14 mg/kg). Group IV 150 mg/kg of APAP, 180 mg/kg of N-acetylcysteine and 280 mg/kg of methionine. The square indicates basal values. Different letters indicate significant differences between the groups (*p*<0.05).

Liver GSH concentration decreased at 4 hours post treatment in groups I, II and IV, while group III (treated with niosomal GSH) showed an increase of GSH liver concentration at this time. In Groups II and III liver GSH concentration remained elevated above the baseline at 24 and 72 hours (Fig. 5).

**Fig. 6:**
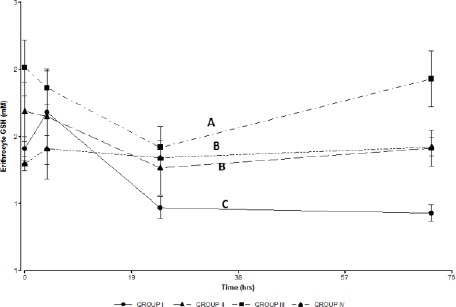
GSH concentration in erythrocytes in the treated groups. Group I: 150 mg/kg of APAP. Group II 150 mg/kg of APAP and GSH 200 mg/kg. Group III 150 mg/kg of APAPand niosomal GSH (14 mg/kg). Group IV 150 mg/kg of APAP, 180 mg/kg of N-acetylcysteine and 280 mg/kg of methionine. The square indicates basal values. Different letters indicate significant differences between the groups (p <0.05).

Group I showed a significant decrease in GSH concentration in the erythrocyte, which remained below the basal levels at 72 hours (Fig. 6) when compared to groups II, III and IV.

## Discussion

Clinical symptoms of APAP intoxication in cats include rapid and significant methemoglobin formation (Nash *et al.*, 1984). Furthermore, oxidation of hemoglobin leads to the denaturation of hemoglobin whose precipitation results in Heinz body formation and hemolytic anemia (Nash *et* *al*., 1984; McConkey *et al.*, 2009). Methemoglobin and hemolytic anemia result in respiratory distress, depression, and weakness that can lead to the death of the animal even prior to the development of liver toxicity.

GSH treatment at 200 mg/kg by intravenous infusion and treatment with niosomal GSH at 14mg/kg by intravenous infusion were found to be effective in decreasing the levels of methemoglobin formation and hepatotoxic effects of APAP poisoning (Fig. 2 and 3).

Treatment with a single oral dose of N-acetyl cysteine and methionine was not effective and did not show any significant differences when compared to the control group (Fig. 2 and 3). This treatment therefore would not be effective against APAP intoxication at a dose of 150 mg/kg. Groups II and III showed a decreased formation of methemoglobin. Both groups showed significant differences from group I and group IV (Fig. 3).

The percentage of methemoglobin was lower in the group that was treated with free GSH (group II). The formation of methemoglobin during intoxication with APAP is a distinctive feature of this poisoning in both dogs and cats (MacNaughton, 2003). Methemoglobin formation occurs by the reaction of NAPQI with the thiol-reactive groups of the hemoglobin molecule (Nash *et al.*, 1984; Savides *et al.*, 1984). The high GSH concentration found in the liver of both species could inactivate NAPQI formed in this organ after APAP administration and thus would reduce the formation of methemoglobin. Additionally, elevated GSH plasma concentrations found at 4 hours post treatment in groups II and III could inactivate NAPQI in plasma, and contribute to reduce methemoglobin formation. Although NAPQI has been proposed as the molecule responsible for the formation of methemoglobin by most authors, McConkey *et al*. (2009) underestimate the importance of NAPQI in the formation of methemoglobin. They propose that the metabolite *para*-aminophenol, which is co-oxidized with the hemoglobin molecule, is more important than NAPQI. However, it is important to note that both the metabolites (NAPQI and *para*-aminophenol) are inactivated by GSH (McConkey *et al.*, 2009).

The hepato-protective effect of GSH in groups II and III would be related to an increase in the GSH hepatic concentration in both groups after treatment. The GSH-induced mechanism of hepatoprotection involves improving the capacity of the hepatocytes to inactivate NAPQI, avoiding the covalent modification of cellular proteins and blocking the initiation of APAP toxicity (Corcoran and Wong, 1986). Free GSH administered intravenously is rapidly degraded by the organs that contain the enzyme γ-glutamyl transpeptidase (γGT). The amino acids released by GSH hydrolysis are absorbed by hepatocytes and subsequently utilized for *de novo* GSH synthesis (Saito *et al.*, 2010). APAP poisoning leads to decreased hepatic concentrations of GSH.

Accordingly, treatment with GSH would be an effective solution because it provides the amino acids that contribute to restoration of liver GSH concentrations (Saito *et al.*, 2010; Masubuchi *et al.*, 2011), and inactivate the NAPQI formed during the metabolic phase of APAP intoxication.

In addition to the hepatoprotective effect of GSH in the metabolic phase of APAP intoxication, there would also be an effect of the treatment during the post metabolic phase. The covalent binding of NAPQI to mitochondrial proteins is a critical point that connected the metabolic phase of APAP toxicity to mitochondrial dysfunction and initiates the post metabolic phase of intoxication (Jaeschke *et al.*, 2011).

This mitochondrial dysfunction leads to inhibition of mitochondrial respiration with the consequent formation of reactive oxygen species and formation of peroxynitrite in the mitochondrial matrix –an effect that consumes mitochondrial GSH (Jaeschke *et al.*, 2011).

The elevated GSH liver concentrations observed in the group treated with free GSH (group II) would help to accelerate the restoration of mitochondrial GSH levels and inactivate the reactive species formed at this stage, thereby limiting hepatocellular necrosis. These effects are associated with a smaller increase in ALT levels in group II when compared to group I.

Hepatoprotective effects were observed in the group treated with niosomal GSH (group III). While the results between groups II and III showed no significant differences, ALT activities were lower in the group treated with niosomes (group III) when compared to the group treated with free GSH (group II).

The group treated with niosomal GSH had a marked increase in hepatic GSH concentration which was significantly different from all other treatments. This finding could be related to the selective accumulation of niosomal GSH in the liver (Uchegbu and Florence, 1995; Hashim *et al.*, 2010).

The niosomal vesicles have a preferential passive targeting in the liver (Azmin *et al.*, 1985; Baillie *et al.*, 1985; Uchegbu and Florence, 1995). After intravenous administration, the vesicles of a size greater than 200 nm are rapidly removed from circulation because, after opsonisation, niosomes are recognized and phagocytosed by Kupffer cells of the liver (Ishida *et al.*, 2002; Mozafari, 2006). Vesicles of 400 nm can also access the hepatocytes depending on the lipid composition and their membrane electric charge (Romero *et al.*, 1999).

Group IV (treated with a combination of amino acids methionine 180mg/kg and N- acetylcysteine 280 mg / kg) showed no significant differences respect to the group I (control). Several authors suggest the use of N-acetylcysteine (NAC) for the treatment of APAP intoxication in cats (Gaunt *et al.*, 1981; Savides *et al.*, 1985) and NAC is also used for the treatment of poisoning in humans (Shen *et al.*, 2011). Thus, our results do not concur with previously published data. One major reason for the observed discrepancy may be related to the differences in the dosing schedule. Gaunt *et al*. (1981) used an oral dosing regimen at 200 mg/kg of NAC every two hours in cats that were intoxicated with 145 mg/kg of APAP and observed significant differences in the formation of methemoglobin respect to the control and found no increase in plasmatic ALT activity.

Savides *et al*. (1985), using oral and intravenous doses of NAC in animals treated with 120 mg/kg of APAP, found both routes of the treatment to be effective, reporting a decrease in the formation of methemoglobin and increased erythrocyte GSH concentration. In a more recent report, Avizeh *et al*. (2010) treated a group of cats with a single dose of NAC (100 mg/kg) along with the administration of 150 mg/kg of APAP and their results agree with those obtained by Gaunt *et al*. (1981).

In conclusion, our results have shown that both free GSH (200 mg/kg) and niosomal GSH (14 mg/kg) treatments were highly effective in reducing both hepatotoxicity and hematotoxicity in cats that were intoxicated with a dose of 150 mg/kg APAP. Niosomes are a novel drug delivery system which offers advantages over other conventional vesicular delivery systems.

Our results show that treatment with niosomal GSH represents an effective therapeutic approach for APAP poisoning. Our results were obtained despite using a lower dose of GSH. This clearly demonstrates that the encapsulated drug is selectively transported to the liver.

Our findings can be of immense help in the treatment of pathophysiological conditions that result in oxidative stress in the liver. From our work emerges the potential for the development of a combined therapy that employs both niosomal and free GSH. Our results suggest that this therapy is more promising than those currently used for the treatment of APAP-induced toxicity.
